# Prevalence and determinants of disability among the adult population aged 18+ years in Somalia evidence from the 2022 Somali Integrated Households Budget Survey

**DOI:** 10.3389/fpubh.2026.1779324

**Published:** 2026-04-30

**Authors:** Jama Barre Hared, Hassan Abdi Ahmed, Abdimalik Ali Warsame

**Affiliations:** 1 Department of Statistics and Data Analytics, Center for Graduate Studies, Jamhuriya University of Science and Technology (JUST), Mogadishu, Somalia; 2 Somalia National Bureau of Statistics, Mogadishu, Somalia; 3 Department of Economics, Faculty of Economics and Management Science, Hormuud University, Mogadishu, Somalia

**Keywords:** adult population, disability, prevalence, SIHBS, Somalia

## Abstract

**Background:**

Notwithstanding disability being a critical public health and social issue in Somalia, available evidence on its magnitude and associated factors remains scarce. Therefore, this study seeks to assess the prevalence of disability and identify its determinants among the adult population aged 18 years and above in Somalia.

**Method:**

A cross-sectional study approach was employed to measure the prevalence of disability and evaluate associated factors. The analysis utilised data from the Somali Integrated Household Budget Survey 2022, with a total sample of 19,832 adult population. A multivariable logit model was applied to evaluate factors influencing disability status. Associations were quantified using adjusted odds ratios (AOR) with 95% confidence interval (CI), and statistical significance was determined at a *p*-value threshold of < 0.05.

**Findings:**

This study found that the overall prevalence of the adult population in Somalia was 11.95% [0.114–0.126]. Women’s disability was marginally higher at 13.29% compared to men’s at 10.36%. Disability was significantly associated with age, sex, education, household size, working status, smoking, residence, and region (*p* < 0.05). Odds increased with age, reaching a peak among older adults at ≥61 years (AOR = 10.700, 95% CI [7.700, 14.860]). Females had higher odds (AOR = 1.440, 95% CI [1.180, 1.750]). Education was protective, higher education (AOR = 0.450, 95% CI [0.330, 0.630]). Non-smokers (AOR = 0.300, 95% CI [0.220, 0.400]) and rural residents (AOR = 0.680, 95% CI [0.550, 0.850]) had lower odds, with notable regional disparities.

**Conclusion:**

Disabilities related to seeing and walking or climbing were the most prevalent in the population. In this regard, efforts should focus on strengthening disability legal frameworks, expanding community screening and mobile health services, implementing gender-responsive and inclusive education programmes, providing targeted social assistance to vulnerable households, and promoting integrated disability care and advancing evidence through multi-survey and comparative research.

## Introduction

Disability is a human condition (1). Disability is not a rare or fixed condition, as many people may encounter functional limitations during their lifetime, and older people will face difficulty in functioning as their age grows (2). Every extended family in Somalia has a person with a disability, and those without impairments are responsible for providing care for them effect (3). Individuals who have lived with a disability are excluded from everyday activities, and people with disabilities face several obstacles when it comes to disability-related services, including employment, education, and access to healthcare (4).

According to the International Classification of Functioning, Disability, and Health (ICF), disability is defined as a broad term that encompasses various conditions, including impairment, constraints on physical activity, and limitations on participation in activities (5). Access to freedom and fundamental rights is emphasized by the Global legal instrument on disability rights adopted by the United Nations (6). Additionally, the Washington group defined disability as any limitation, such as psychological or physical issues, or difficulty in performing Activities of Daily Living (ADL), that could hinder social interactions with the individual’s living environment. The Washington organization defines disability as having trouble with one or more of the following: hearing, seeing, walking, climbing stairs, concentrating, and remembering (7). The World Health Organization (WHO) estimated that 15% of the world’s population has some form of disability, the prevalence of disability worldwide was estimated to be 14% based on data from the World Healthy Survey (WHS). However, the analysis of measuring disability may have disparities, depending on the research method used in data collection, reporting method, research questions deployed, and the definitions of the variables which influence the outcome of the study (6). overall prevalence of disability among people in Somalia aged 18 and over is 11.7. This indicates that the number is crucial to consider when developing policies and programs that can improve their well-being (3).

Despite the considerable number, people with disabilities continue to face significant challenges throughout their lives (8, 9). These difficulties include being excluded from society, stigma, lack of access to health care, clean drinking water, basic hygiene services, low school attendance, and limited market access (10). They not only make it difficult for people to participate in decision-making processes that impact their role in society, but they also hinder their social lives (11). These issues have persisted due to a lack of laws and initiatives that enhance and foster social acceptance (7). Equally, People living with disabilities (PLWD) have poorer living conditions than the general population, and they are more vulnerable to age-related health issues, injuries, and noncommunicable chronic illnesses (12).

Research conducted in China revealed that the prevalence of self-care disability markedly affects the older adults, especially among women and individuals living in rural regions (13). In Ethiopia, similar analysis revealed a strong relationship with aging, cognitive limitations, and depression (14). Other studies also indicated that a growing proportion of older adults would experience various difficulties, including issues with sight, hearing, or other types (14). A population-based cross-sectional study in Malaysia indicates that chronic illnesses, low educational attainment, and progressive age significantly influence physical disability and functional weakening (15). These results aligned with the studies conducted in Ethiopia (15). Other studies reported that low educational attainment, chronic illnesses, and poverty are substantial factors contributing to impairment (16). Based on existing literature, this study incorporated a set of demographic and socioeconomic variables to assess the prevalence and contributing determinants of disability in Somalia, including sex (6), age (2), educational attainment (17), marital status (18), household size poverty status (19, 20) employment status (21–23), smoking behavior (14), place of residence (7, 19), and geographic region (24).

In Somalia, the government enacted a law in December 2018 that established the National Disability Agency, to protect disabled population, facilitating access to government services including education, equitable employment opportunities, and healthcare, moreover, the agency’s mandate encompasses providing people with disabilities access to assistive technology for walking and hearing, in addition to human capital development initiatives aimed at enhancing the skills of people with disabilities. This follows the ratification of the UN human rights treaty protecting persons with disabilities by the Somali government (3).

Despite Somalia’s efforts to operationalize national and international frameworks for protecting people with special needs. The existing research on disability in Somalia remains extremely limited. a few studies conducted to date suffer from major shortcomings, including limited data depth, small sample sizes, and outdated publications (25). Moreover, many of these studies adopt a narrow focus, which constrains evidence-based policymaking and hinders the effective design of inclusive interventions at the national level (26). The absence of disaggregated statistics makes it exceedingly challenging to ascertain the accurate number of individuals living with disabilities in Somalia (27).

Therefore, this study explores the extent of disability and the determinants associated with it among adults aged 18 years and above in Somalia, drawing on data from the 2022 Household Budget Survey. The survey’s large and nationally representativeness ensures the robustness and coverage, allowing the generalizability of the findings, to reliably reflect the situation of adults across the country. This study is critical for guiding the formulation of effective policies, strategies and well-targeted interventions aligned with Somalia’s national transformation plan 2025 that outlines the rights and needs of the people with disability (28). As the first national-level analysis to address disability in Somalia, the study provides a unique empirical foundation to support informed, data-driven decision-making by policymakers and development actors.

## Methods and materials

### Study setting

Somalia, the Federal Republic, is positioned in the Horn of Africa and adjacent to the Federal Democratic Republic of Ethiopia, the Republic of Djibouti, and the Republic of Kenya; it also has a sea border with Yemen. It spans almost 637,657 km^2^. Somalia also enjoys the broadest stretch of continental shoreline in Africa, at 3,330 km. The nationally recognized languages of the country include Somali, Arabic and English. It also has two rivers, namely the Juba and Shabelle rivers, coming from the Ethiopian mountains, which are essential for livelihoods (20).

### Sampling and design

This study used secondary data from the 2022 Somali Integrated Household Budget Survey (SIHBS), a nationally representative survey (24). A cross-sectional approach was applied, assessing the prevalence of disability and key associated determinants. The target population was adults aged 18+ years in Somalia (3). The sample size consisted of 19,832 adults drawn from 601 EAs across 17 regions, ensuring both national and regional representation (24). SIHBS employed a stratified multi-stage sampling design covering urban, rural, and nomadic populations (19). In urban and rural areas, a three-stage cluster approach was used: Enumeration Areas (EAs) as Primary Sampling Units selected via probability proportional to size (PPS), followed by household listing and PPS-based household selection (20). Nomadic areas used a comparable two-stage stratified design. From each selected EA, 12 households were randomly sampled after listing and data cleaning (19). Data collection relied on digitally administered structured questionnaires, with interviews conducted primarily with the household head or the most knowledgeable adult (15 years and above) when the head was unavailable, ensuring accuracy and completeness of information collected (3).

### Study variables

#### Outcome

The outcome variable was constructed using responses to six functional domains in the SIHBS 2022 dataset. The six domains were seeing, hearing, walking, remembering, self-care and communication. The measure reflects the maximum level of difficulty an individual experiences from any functional limitation, categorized into five response options: “1 no difficulty” is categorized for not having any form of disability; in contrast, “2 some difficulty,” “3 a lot of difficulty,” and “4 cannot do at all” are grouped for having any form of disability for the purpose of analysis. The outcome was recoded into a binary (1 = No, for those reported “no difficulty” while 2 = Yes, for those answered “some difficulty,” “a lot of difficulty,” or “cannot do at all”) (64). The analysis omitted responses characterized as “5 don’t know” to prevent any misinterpretation of the results. The study was restricted to individuals aged 18 years and older residing in the selected areas. This age criterion is consistent with approaches adopted in similar studies conducted globally (29–31).

#### Independent

Based on existing literature, the following demographic and socioeconomic variables were analyzed, including; sex, age, highest education level, marital status, household size, poverty status, employment, smoking behavior, place of residence, and geographic region. Sex was categorized as male or female (17, 32, 33). Age was divided into five groups (18–30, 31–40, 41–50, 51–60, and 61 years and above) to capture differences across key stages of adulthood (6). Education was classified into four levels: no formal schooling, primary school education, secondary school education, and university education (6). Marital status consisted of four categories: married, divorced, never married, and widowed (34). Household size was grouped into small (1–3 members), medium (4–6 members), and large (7 or more members) (18). Poverty was measured dichotomously as poor or non-poor (20). Employment status distinguished between individuals who were working and those not (35). Smoking behavior was recorded as either yes or no (14). Initially, place of residence was classified as urban, rural, and nomadic; however, to ensure the appropriateness and stability of the logistic regression analysis, rural, and nomadic was subsequently combined into “rural areas” (7, 19). Regional analysis was conducted across the 17 administrative regions captured in the SIHBS dataset: Awdal, Bakool, Banadir, Bari, Bay, Galgaduud, Gedo, Hiraan, Lower Juba, Lower Shabelle, Waqooyi Galbeed, Middle Shabelle, Mudug, Nugaal, Sanaag, Sool, and Togdheer (24). These classifications enabled the study to examine how individual, household, and regional characteristics are associated with variations in disability status.

### Data analysis

Adults aged 18 years and above constituted the unit of analysis for this study. Prior to analyses, the complex survey design of the 2022 Somali Integrated Household Budget Survey (SIHBS) was clearly stated using the svyset command, incorporating sampling weights (wgt), stratification by region, and clustering at the Enumeration Area level (EA), which served as the primary sampling unit (PSU). Variance covariance estimation (VCE) was performed to ensure that standard errors, 95% confidence intervals (CIs), and *p*-values appropriately accounted for the sampling design. Subsequently, descriptive statistics were first generated and summarized using frequencies and percentages. Thereafter, the associations between the outcome variable and explanatory factors were initially assessed using bivariable survey-adjusted logistic regression analysis. Multicollinearity among the independent variables was examined using the Variance Inflation Factor (VIF), and no evidence of problematic multicollinearity was observed, as all VIF values were below the commonly accepted threshold of 5. Variables with a *p*-value of less than 0.25 in the bivariable analysis were considered eligible for inclusion in the multivariable survey-adjusted logistic regression model, and all selected variables satisfied the inclusion criteria (19, 36). In the final multivariable model, associations were reported as adjusted odds ratios (AORs) with corresponding 95% confidence intervals, and statistical significance was determined at *p* < 0.05. All statistical analyses were performed using Stata version 17.

### Findings

Table 1 depicts the overall prevalence of disabilities among the Somali adult population aged 18 and above. In total, 19,832 adults were included in the analysis; 11.95% reported having at least one disability, whereas 88% indicated no disability.

**Table 1:** Prevalence of disability among adult population aged 18+ years in Somalia (*n* = 19,832).

Any disability	*n* = 19,832	Prevalence	Std. err.	[95% confidence interval]
No	17,463	88.05%	0.003	[0.874–0.886]
Yes	2,369	11.95%	0.003	[0.114–0.126]

Table 2 outlines the socioeconomic and demographic information of the respondents, consisting of 19,832 adults aged 18 years and older. Females comprised 54.14%, while males accounted for 45.86%. Adults aged 18–30 years constituted the largest proportion of the study population (42.38%), followed by those aged 31–40 years (23.06%) and 41–50 years (14.00%). In contrast, participants aged 51–60 years and 61 years or older accounted for 8.30 and 12.26%, respectively. In terms of education, 36.39% of the adults had completed primary school education, 31.59% had secondary school education, and 15.39% had university education, while 16.63% reported no formal education. The majority of respondents were married, 60.74%, whereas 25.79% were never married, 6.53% were divorced, and 6.93% were widowed. Household size varied: 47.47% of households had seven or more members, 41.06% were in households with 4–6 members, and 11.47% were in the smallest households of 1–3 members. Most respondents were not working 83.55%, while only 16.45% reported being employed. In terms of economic status, 48.76% of the population was classified as poor, whereas 51.24% was characterized as not poor. A very small proportion of respondents reported smoking, 2.79%, while the vast majority did not smoke, 97.21%. Most of the population lives in urban areas 65.05%, while 34.95% live in rural settings. Regarding the regional distribution, the largest proportion of respondents was from Waqooyi Galbeed (10.16%), followed by Banadir (9.40%). Togdheer accounted for 6.12% of the respondents, while Bari contributed 6.00%. Nugaal, Sanaag, and Lower Shabelle each represented 5.57% of the total sample. The remaining regions each accounted for between 4.82 and 5.72% of respondents, indicating a relatively balanced distribution across the other regions.

**Table 2:** Socioeconomic and demographic profile of the population aged 18+ years in Somalia.

Variable	Category	Weighted count	Weighted (%)
Sex	Male	9,094	45.86
	Female	10,738	54.14
Age	18–30 years	8,405	42.38
	31–40 years	4,573	23.06
	41–50 years	2,776	14.00
	51–60 years	1,647	8.30
	≥ 61 years	2,431	12.26
Highest education level	No formal education	1,372	16.63
	Primary school	3,002	36.39
	Secondary school	2,606	31.59
	University	1,270	15.39
Marital status	Married	12,046	60.74
	Divorced	1,296	6.53
	Never married	5,115	25.79
	Widowed	1,375	6.93
Household size	1–3 members	2,275	11.47
	4–6 members	8,143	41.06
	≥ 7 members	9,414	47.47
Working status	Working	3,262	16.45
	Not working	16,570	83.55
Poverty status	Not poor	10,162	51.24
	Poor	9,670	48.76
Smoking status	Yes	554	2.79
	No	19,278	97.21
Residence	Urban	12,900	65.05
	Rural	6,932	34.95
Region	Awdal	1,134	5.72
	Bakool	989	4.99
	Banadir	1,864	9.40
	Bari	1,190	6.00
	Bay	955	4.82
	Galgaduud	1,039	5.24
	Gedo	1,044	5.26
	Hiraan	998	5.03
	Lower Juba	978	4.93
	Lower Shabelle	1,104	5.57
	Waqooyi Galbeed	2,015	10.16
	Middle Shabelle	1,027	5.18
	Mudug	1,010	5.09
	Nugaal	1,104	5.57
	Sanaag	1,104	5.57
	Sool	1,063	5.36
	Togdheer	1,214	6.12

**Figure 1:**
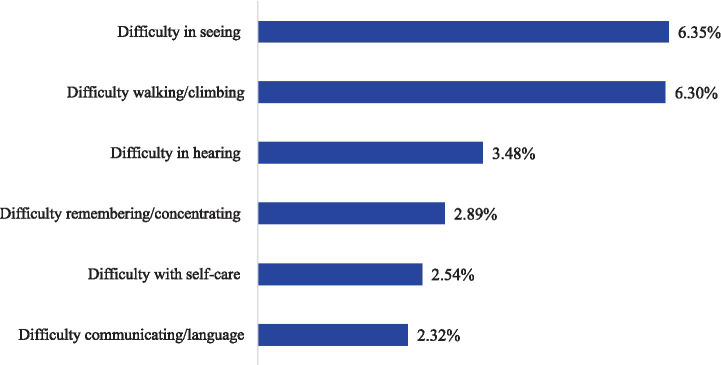
Distribution of disability types among the adult population aged 18+ years in Somalia.

Figure 1 displays the distribution of disability types among the study population (aged 18+ years) in Somalia. Difficulties related to seeing 6.35%, and walking or climbing 6.30% were the most prevalent disabilities. In contrast, communication or language-related difficulties represented the least prevalent form of disability in the population.

Table 3 displays the bivariate association between disability status and selected socioeconomic and demographic characteristics among the adult population in Somalia. The selected predictors were sex, age, highest education level, marital status, household size, poverty, working and smoking status, as well as residence and region of the study population. Overall, disability was significantly associated with all independent variables (*p* < 0.001).

**Table 3:** Bivariate analysis of disability across socioeconomic and demographic characteristics.

Variable	Any disability				*p*-value	
	No		Yes			
	%	[95% CI]	%	[95% CI]	Freq	
Sex						*p* < 0.001
Male	89.64	[0.890, 0.903]	10.36%	[0.097, 0.110]	9,094	
Female	86.71	[0.861, 0.873]	13.29%	[0.127, 0.139]	10,738	
Age						*p* < 0.001
18–30 years	95.67	[0.952, 0.960]	4.33%	[0.039, 0.047]	8,405	
31–40 years	92.94	[0.921, 0.936]	7.06%	[0.063, 0.078]	4,573	
41–50 years	86.71	[0.853, 0.879]	13.29%	[0.120, 0.146]	2,776	
51–60 years	79.78	[0.777, 0.816]	20.22%	[0.183, 0.222]	1,647	
> = 61 years	59.69	[0.577, 0.616]	40.31%	[0.383, 0.422]	2,431	
Education						*p* < 0.001
No formal	90.31	[0.886, 0.917]	9.69%	[0.082, 0.113]	1,372	
Primary school	92.04	[0.910. 0.929]	7.96%	[0.070, 0.089]	3,002	
Secondary school	94.09	[0.931, 0.949]	5.91%	[0.050, 0.068]	2,606	
University	94.72	[0.933, 0.958]	5.28%	[0.041, 0.066]	1,270	
Marital status						*p* < 0.001
Married	88.65	[0.880, 0.892]	11.35%	[0.107, 0.119]	12,046	
Divorced	86.73	[0.847, 0.884]	13.27%	[0.115, 0.152]	1,296	
Never married	94.70	[0.940, 0.952]	5.30%	[0.047, 0.059]	5,115	
Widowed	59.35	[0.567, 0.619]	40.65%	[0.380, 0.432]	1,375	
Household size						*p* < 0.001
1–3 members	88.35	[0.869, 0.896]	11.65%	[0.103, 0.130]	2,275	
4–6 members	88.19	[0.874, 0.888]	11.81%	[0.111, 0.125]	8,143	
> = 7 members	87.87	[0.871, 0.885]	12.13%	[0.114, 0.128]	9,414	
Poverty status						*p* < 0.001
Not poor	87.84	[0.871, 0.884]	12.16%	[0.115, 0.128]	10,162	
poor	88.28	[0.876, 0.889]	11.72%	[0.110, 0.123]	9,670	
Working status						*p* < 0.001
Working	90.44	[0.893, 0.914]	9.56%	[0.086, 0.106]	3,262	
Not working	87.59	[0.870, 0.880]	12.41%	[0.119, 0.129]	16,570	
Smoking						*p* < 0.001
Yes	88.81	[0.859, 0.911]	11.19%	[0.088, 0.141]	554	
No	88.03	[0.875, 0.884]	11.97%	[0.115, 0.124]	19,278	
Residence						*p* < 0.001
Urban	88.26	[0.877, 0.888]	11.74%	[0.111, 0.123]	12,900	
Rural	87.67	[0.868, 0.884]	12.33%	[0.115, 0.131]	6,932	
Region						*p* < 0.001
Awdal	83.77	[0.815, 0.858]	16.23%	[0.815, 0.858]	1,134	
Bakool	86.25	[0.839, 0.882]	13.75%	[0.839, 0.882]	989	
Banadir	89.11	[0.876, 0.904]	10.89%	[0.876, 0.904]	1864	
Bari	85.29	[0.831, 0.871]	14.71%	[0.831, 0.871]	1,190	
Bay	87.43	[0.851, 0.893]	12.57%	[0.851, 0.893]	955	
Galgaduud	88.35	[0.862, 0.901]	11.65%	[0.862, 0.901]	1,039	
Gedo	88.41	[0.863, 0.902]	11.59%	[0.863, 0.902]	1,044	
Hiraan	90.28	[0.882, 0.919]	9.72%	[0.882, 0.919]	998	
Lower Juba	94.89	[0.933, 0.961]	5.11%	[0.933, 0.961]	978	
Lower Shabelle	94.02	[0.924, 0.952]	5.98%	[0.924, 0.952]	1,104	
Waqooyi Galbeed	88.34	[0.868, 0.896]	11.66%	[0.868, 0.896]	2015	
Middle Shabelle	87.05	[0.848, 0.889]	12.95%	[0.848, 0.889]	1,027	
Mudug	90.20	[0.882, 0.918]	9.80%	[0.882, 0.918]	1,010	
Nugaal	88.86	[0.868, 0.905]	11.14%	[0.868, 0.905]	1,104	
Sanaag	84.42	[0.821, 0.864]	15.58%	[0.821, 0.864]	1,104	
Sool	85.51	[0.832, 0.875]	14.49%	[0.832, 0.875]	1,063	
Togdheer	85.17	[0.830, 0.870]	14.83%	[0.830, 0.870]	1,214	

**Figure 2:**
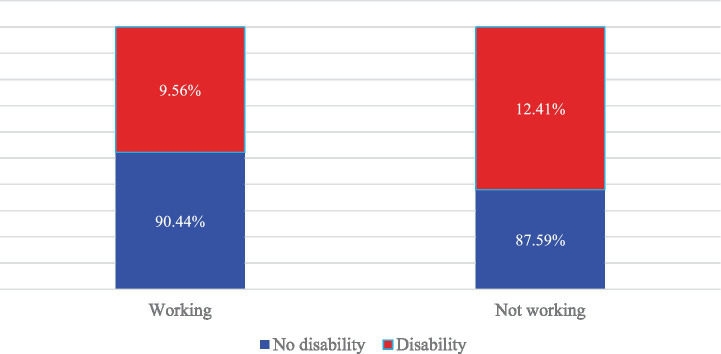
Distribution of working status by disability among the study population (%).

Figure 2 displays the distribution of working status by disability among the study population (aged 18+ years) in Somalia. The proportion of persons with disabilities was higher among those who were not working in the past week (12.41%) compared with those who were working (9.56%). In contrast, individuals without disabilities constituted the majority in both groups, accounting for 90.44% of those working and 87.59% of those not working, indicating a modest disparity in employment participation by disability status.

Table 4 summarizes the multicollinearity diagnostic results based on the Variance Inflation Factor (VIF) analysis. VIF statistics of the predictors included in the regression model. All VIF values ranged from 1.01 (region) to 1.31 (age and marital status), with a mean VIF of 1.11. Since all VIF values were well below the commonly accepted threshold of 5, multicollinearity was not considered a concern in this analysis. Furthermore, tolerance values (1/VIF) were all above 0.70, confirming that the independent variables do not exhibit problematic levels of collinearity.

**Table 4:** Multicollinearity test.

Variable	VIF	1/VIF
Sex	1.07	0.932953
Age	1.31	0.763854
Highest education level	1.15	0.872730
Marital Status	1.31	0.763231
Household size	1.05	0.952154
Working status	1.02	0.983237
Poor status	1.05	0.947894
Smoke	1.04	0.963759
Residence	1.10	0.905977
Region	1.01	0.985554
**Mean VIF**	**1.11**	**—**

Mean VIF, the average of the variance Inflation factors (VIFs) across all predictors in a regression model.

Table 5 displays a multivariable logit regression model was conducted to examine determinants of disability among adult population in Somalia. The following variables age, sex, education, household size, working, and smoking status as well as residence and region were found to be statistically significant predictors of disability at *p* < 0.05. Age was the strongest determinant of disability. Compared to adults aged 18–30 years (reference), the odds of disability increased substantially with age. Respondents aged 41–50 years had more than twice the odds of being disabled (AOR = 2.54, 95% CI [1.91, 3.39], *p* < 0.001), while those aged 51–60 years had nearly five times higher odds (AOR = 4.98, 95% CI [3.64, 6.81], *p* < 0.001). Similarly, those aged 61 years and older were approximately eleven times more likely to have experienced disability (AOR = 10.70, 95% CI [7.70, 14.86], *p* < 0.001). Regarding, Sex of the respondent was also significant, with females having higher odds ratio of disability than males (AOR = 1.44, 95% CI [1.18, 1.75], *p* < 0.001). Furthermore, higher levels of education appeared to reduce the likelihood of disability. Additionally, education demonstrated a protective effect. Compared to those with no formal education, adults with primary school (AOR = 0.65, 95% CI [0.52, 0.81], *p* < 0.001), secondary school (AOR = 0.56, 95% CI [0.44, 0.72], *p* < 0.001), and university (AOR = 0.45, 95% CI [0.33, 0.63], *p* < 0.001) had significantly lower odds of reporting disability. Household size showed a moderate association. Individuals in households with seven or more members had reduced odds of disability compared to those in households with 1–3 members (AOR = 0.75, 95% CI [0.58, 0.97], *p* < 0.01). Working status was significantly associated with disability, with individuals who were not working exhibiting slightly lower odds of reporting disability compared with those who were employed (AOR = 0.80, 95% CI [0.65, 0.99], *p* < 0.01). Smoking was strongly associated with disability, with non-smokers less likely to report disability when compared to smokers (AOR = 0.30, 95% CI [0.22, 0.40], *p* < 0.001). Place of residence showed a significant effect, with rural residents having lower odds of disability than those in urban areas (AOR = 0.68, 95% CI [0.55, 0.85], *p* < 0.001). Regional disparities were also notable. Compared to the Awdal region (reference), adults residing in Banadir (AOR = 0.34, 95% CI [0.24, 0.48], *p* < 0.001), Bakool (AOR = 0.49, 95% CI [0.32, 0.75], *p* < 0.001), Lower Juba (AOR = 0.12, 95% CI [0.06, 0.25], *p* < 0.001), Middle Shabelle (AOR = 0.07, 95% CI [0.02, 0.19], *p* < 0.001), and other regions showed significantly lower odds of disability.

**Table 5:** Multivariable logistic regression of determinants associated with disability among the adult population in Somalia.

Variable	Category	Odds ratio	[95% CI]	*p*-value
Sex (ref: male)	Female	1.438	[1.180, 1.753]	*p* < 0.001
Age (ref: 18–30 years)	31–40 years	1.223	[0.933, 1.604]	0.144
	41–50 years	2.544	[1.908, 3.391]	*p* < 0.001
	51–60 years	4.978	[3.640, 6.809]	*p* < 0.001
	≥ 61 years	10.698	[7.701, 14.86]	*p* < 0.001
Marital status (ref: married)	Divorced	1.185	[0.832, 1.686]	0.346
	Never married	0.814	[0.625, 1.060]	0.128
	Widowed	1.252	[0.827, 1.893]	0.287
HH size (ref: 1–3 members)	4–6 members	0.920	[0.714, 1.185]	0.522
	≥ 7 members	0.745	[0.575, 0.965]	*p* < 0.01
Smoking (ref: yes)	No	0.295	[0.217, 0.401]	*p* < 0.001
Highest education level (ref: no formal)	Primary school	0.651	[0.521, 0.813]	*p* < 0.001
	Secondary school	0.562	[0.438, 0.722]	*p* < 0.001
	University	0.454	[0.330, 0.625]	*p* < 0.001
Working status (ref: working)	Not working	0.801	[0.650, 0.986]	*p* < 0.01
Poverty status (ref: not poor)	Poor	0.936	[0.776, 1.127]	0.487
Residence (ref: urban)	Rural	0.681	[0.549, 0.845]	*p* < 0.001
Region (ref: Awdal)	Bakool	0.491	[0.322, 0.748]	*p* < 0.001
	Banadir	0.335	[0.235, 0.476]	*p* < 0.001
	Bari	0.336	[0.231, 0.491]	*p* < 0.001
	Bay	0.311	[0.192, 0.506]	*p* < 0.001
	Galgaduud	0.305	[0.177, 0.525]	*p* < 0.001
	Gedo	0.287	[0.172, 0.478]	*p* < 0.001
	Hiraan	0.324	[0.212, 0.497]	*p* < 0.001
	Lower Juba	0.117	[0.055, 0.250]	*p* < 0.001
	Lower Shabelle	0.145	[0.085, 0.247]	*p* < 0.001
	Waqooyi Galbeed	0.276	[0.195, 0.391]	*p* < 0.001
	Middle Shabelle	0.066	[0.023, 0.188]	*p* < 0.001
	Mudug	0.258	[0.152, 0.437]	*p* < 0.001
	Nugaal	0.382	[0.246, 0.593]	*p* < 0.001
	Sanaag	0.330	[0.218, 0.499]	*p* < 0.001
	Sool	0.368	[0.250, 0.543]	*p* < 0.001
	Togdheer	0.301	[0.195, 0.464]	*p* < 0.001

## Discussion

Disability is a social phenomenon that restricts the persons’ ability. In Somalia, 11.95% [0.114–0.126] of the adult population aged 18 and older have experienced at least one type of disability. In this study, vision 6.35% and mobility 6.30% difficulties were the most common types of disability among the population. This finding shows that functional disabilities are a major challenge for the Somali population aged 18+, underscoring the importance of effective health-care planning to address and prevent these limitations. This study aligns with the report from Somalia (3) and mirrors a study conducted in South Africa (37–39). The alignment between the studies could be attributed to their comparable research approach and the equivalent average ages of respondents.

Demographic variables including sex, age, household size and smoking status significantly impact the prevalent of disability. The findings of our analysis indicates that females had 1.438 times greater odds of disability than their male counterparts (AOR = 1.438; 95% CI [1.180–1.753]), demonstrating a significant relationship between sex and disability. This finding is conformed with earlier study (40–44). The study also demonstrated a significant association between age and disability. Adults aged 41–50 years exhibited substantially higher odds of experiencing disability (AOR = 2.544, 95% CI [1.908, 3.391]), suggesting 2.544 times more likely to have at least difficulties compared with those aged 18–30 years. Likewise, individuals aged 51–60 years were found to have nearly five times greater odds of disability relative to the 18–30 age group (AOR = 4.978, 95% CI [3.640, 6.809]). Furthermore, adults aged 61 years and above had markedly increased odds of disability, approximately ten times higher than their younger counterparts (AOR = 10.698, 95% CI [7.701, 14.860]). These results indicate a strong, progressive age-related increase in the likelihood of disability. This corresponds with worldwide research identifying ageing as a central determinant of functional impairment, influenced by chronic health conditions, increased frailty, and limited provision of assistive equipment (17, 18, 45, 46).

In household size, the analysis revealed that larger households had lower odds of experiencing disability (AOR = 0.745, 95% CI [0.575, 0.965]) compared to households with 1–3 members. One possible explanation is that households with more members may provide stronger social support networks and greater availability of caregiving assistance. This result is consistent with evidence reported in earlier studies (18, 47, 48). In contrast, research from high-income countries often shows the opposite pattern, where larger household size is associated with increased disability risk due to heightened economic pressures and resource limitations (49). Similarly, the study explained that non-smokers had significantly lower odds of disability (AOR = 0.295, 95% CI [0.217, 0.401]). This indicates that adults who do not smoke were about 70% less likely to report disability compared with those who smoke. This finding is consistent with the established evidence that smoking contributes to chronic conditions and functional decline, whereas non-smoking is protective for overall health and functional (50–52). Given that smoking status was self-reported, underreporting is reasonably plausible, particularly in the Somali context where restrictive social norms may limit disclosure of smoking behavior. This may partly explain the observed protective association and therefore justifies cautious interpretation of the finding.

Moreover, Education underscored an inverse association with disability, with higher schooling linked to progressively lower odds of experiencing functional difficulties. Those with primary school education had less disability risk compared with those with no formal schooling (AOR = 0.651, 95% CI [0.521–0.813]). This protective effect strengthened at higher levels of education: those with secondary school education had lower odds (AOR = 0.562, 95% CI [0.438–0.722]), while individuals with university education experienced the greatest reduction, with lower odds of disability (AOR = 0.454, 95% CI [0.330–0.625]). This pattern highlights the role of education in reducing vulnerability to disability, where higher education is linked better job opportunity enhanced healthy literacy and reduced exposure with disability. This finding is aligned by other studies conducted globally (53–55). In contrast, evidence from high-income countries with well-established social protection systems, such as the United States, shows a much weaker association between education and disability. This is largely because government welfare programs and income support reduce the socio-economic disparities that typically magnify disability risks among individuals with lower levels of education (56, 57). Working status was significantly associated with disability, as individuals who were not employed had lower odds of reporting disability compared with their counterparts (AOR = 0.801, 95% CI [0.650, 0.986]). This result is corroborated with previous studies (21–23). This association may reflect broader labour market conditions, where persons with disabilities often face barriers to obtaining or maintaining employment, rather than indicating any protective effect of non-employment. In regarding, the residence emerged as an important factor. In Somalia respondents living in rural areas had considerably lower odds of disability than their urban counterparts (AOR = 0.681, 95% CI [0.549, 0.845], *p* < 0.001). This finding aligned other studies conducted in countries like Afghanistan (58). Difference may reflect variations in daily activities, environmental exposures, diagnostic availability, or reporting behavior, as urban populations often have greater access to health services and may therefore identify disabilities more readily. Collectively, these results underscore how socio-economic and geographic contexts shape the distribution and reporting of disability. However multiple studies contradict the findings of this research arguing that, disability is more prevalent in urban areas compare to rural settings (59, 60).

Lastly, the study revealed considerable regional disparities in disability status. Awdal was used as the reference category based on the default coding structure of the dataset; Therefore, the choice of reference category (Awdal) may influence the interpretation of the regional Adjusted Odds Ratios. The sharpest declines were seen in Middle Shabelle (AOR = 0.066, 95% CI [0.023, 0.188]) and Lower Juba (AOR = 0.117, 95% CI [0.055, 0.250]), indicating very low reported disability in these areas. Regions such as Bay (AOR = 0.311, 95% CI [0.192, 0.506]), Galgaduud (AOR = 0.305, 95% CI [0.177, 0.525]), and Waqooyi Galbeed (AOR = 0.276, 95% CI [0.195, 0.391]) also showed substantially reduced odds. More moderate decreases were noted in Bakool (AOR = 0.491, 95% CI [0.322, 0.748]), Nugaal (AOR = 0.382, 95% CI [0.246, 0.593]), and Sool (AOR = 0.368, 95% CI [0.250, 0.543]) (61–63). Overall, these findings highlight significant geographic disparities, suggesting that regional differences in context, population characteristics, and service accessibility shape the distribution and reporting of disability across Somalia.

### Strengths and limitations

This study draws upon nationally representative data, offering a strong and credible basis for estimating disability prevalence among adults in Somalia. The survey utilized internationally standardized and validated data collection tools, which enhances the consistency, comparability, and reliability of the findings. Disability was assessed across six key functional domains: seeing, hearing, remembering, walking, self-care, and communication/language to provide a comprehensive understanding of the functional limitations experienced by older adults. It adds valuable evidence to the limited body of literature on disability and its associated factors. In assessing socioeconomic status, poverty status was used instead of wealth quintiles due to the absence of wealth-related variables in the dataset. However, several limitations should be noted. As a cross-sectional study, it cannot establish causal relationships between disability and the associated factors identified. Depending on self-reported functional difficulties, without clinical verification, may introduce reporting prejudice. On top of that, important variables such as cooking fuel and chronic health conditions (including diabetes, hypertension, hepatitis, and anaemia) were not included due to data gaps, although these factors may influence disability outcomes among older adults. Despite these challenges, the study contributes significantly to addressing the existing knowledge gap regarding disability among older populations in Somalia.

### Recommendation

This study underscores age, sex, education level, household size, employment status, smoking behavior, residence, and region as key determinants of disability among adults in Somalia. The following recommendations are proposed: First, the government should lay down legal frameworks, policies, and strategies on disability to improve the socio-economic and demographic status of people with disabilities. Second, the government and health sector stakeholders should expand community-based screening programs targeting older adults and provide mobile health clinics in underserved and rural areas to improve access to early diagnosis and functional assessments to integrate care for older adults into primary health services, particularly for chronic disease management. Third, the National Disability Agency, with the Ministry of Health, should develop programs that respond to the specific needs of women and men with disabilities to ensure women with disabilities have access to healthcare, livelihood opportunities, and protection services, considering their heightened vulnerability. Fourth, the Ministry of Education, Culture, and Higher Education should continue strengthening initiatives such as the Inclusive Education Policy, Accelerated Basic Education, and Alternative Basic Education programs to expand adult literacy programs and inclusive education initiatives. Finally, the government and its partners should provide social assistance (cash transfers, food support, and health subsidies) for large or economically strained households caring for persons with disabilities. Although this study provides the first nationally representative evidence for Somalia, the determinants identified are broadly consistent with patterns well established in other low- and middle-income countries. Therefore, the study’s principal contribution lies in filling a critical geographic evidence gap for Somalia. Additionally, future research should incorporate multi-survey datasets and comparative analysis across different national contexts to generate more profound insights.

## Data Availability

The datasets presented in this study can be found in online repositories. The names of the repository/repositories and accession number(s) can be found at: https://microdata.nbs.gov.so/index.php/catalog/59/get-microdata.
