# Supplementary material for: Prevalence and determinants of disability among the adult population aged 18+ years in Somalia evidence from the 2022 Somali Integrated Households Budget Survey
Source: Front Public Health. 2026 Apr 30;14:1779324. doi: 10.3389/fpubh.2026.1779324 (PMC13171764; doi:10.3389/fpubh.2026.1779324)
Supplement: Supplementary file 1 [file Data_Sheet_1.pdf]

Inferential statistics

Table S. **Estimates of Unadjusted and Adjusted Odds Ratio.**

Variable	Unadjusted OR	[95% CI]	p-value	Adjusted OR	[95% CI]	p-value
**Sex**						
Male	1			1		
Female	1.33	[1.22–1.45]	<0.001	1.438	[1.180, 1.753]	0.001
**Age**						
18–30 years	1			1		
31–40 years	1.68	[1.44–1.96]	<0.001	1.223	[0.933, 1.604]	0.144
41–50 years	3.39	[2.91–3.94]	<0.001	2.544	[1.908, 3.391]	0.001
51–60 years	5.60	[4.77–6.57]	<0.001	4.978	[3.640, 6.809]	0.001
≥ 61 years	14.92	[13.07–17.04]	<0.001	10.698	[7.701, 14.86]	0.001
**Marital status**						
Married	1			1		
Divorced	1.20	[1.01–1.42]	0.040	1.185	[0.832, 1.686]	0.346
Never married	0.44	[0.38–0.50]	<0.001	0.814	[0.625, 1.060]	0.128
Widowed	5.35	[4.74–6.04]	<0.001	1.252	[0.827, 1.893]	0.287
Household size						
1–3 members	1			1		
4–6 members	1.02	[0.88–1.17]	0.829	0.920	[0.714, 1.185]	0.522
≥ 7 members	1.05	[0.91–1.21]	0.526	0.745	[0.575, 0.965]	0.01
**Smoking**						
Yes	1			1		
No	1.08	[0.83–1.41]	0.579	0.295	[0.217, 0.401]	0.001
**Education**						
No formal	1			1		
Primary school	0.81	[0.65–1.01]	0.057	0.651	[0.521, 0.813]	0.001
Secondary school	0.59	[0.46–0.75]	<0.001	0.562	[0.438, 0.722]	0.001
University	0.52	[0.38–0.70]	<0.001	0.454	[0.330, 0.625]	0.001
**Working status**						
Working	1			1		
Not working	0.26	[0.076-0.45]	0.006	0.801	[0.650, 0.986]	0.01
**Poverty status**						
Not poor	1			1		
Poor	0.96	[0.88–1.04]	0.333	0.936	[0.776, 1.127]	0.487
**Residence**						
Urban	1.00			1		
Rural	1.06	[0.97–1.16]	0.216	0.681	[0.549, 0.845]	0.001
**Region**						
Awdal	1					
Bakool	0.82	[0.65–1.05]	0.112	0.491	[0.322, 0.748]	0.001
Banadir	0.63	[0.51–0.78]	0.001	0.335	[0.235, 0.476]	0.001
Bari	0.89	[0.71–1.11]	0.311	0.336	[0.231, 0.491]	0.001
Bay	0.74	[0.58–0.95]	0.018	0.311	[0.192, 0.506]	0.001

Galgaduud	0.68	[0.53–0.87]	0.002	0.305	[0.177, 0.525]	0.001
Gedo	0.68	[0.53–0.87]	0.002	0.287	[0.172, 0.478]	0.001
Hiraan	0.56	[0.43–0.72]	0.001	0.324	[0.212, 0.497]	0.001
Lower Juba	0.28	[0.20–0.39]	0.001	0.117	[0.055, 0.250]	0.001
Lower Shabelle	0.33	[0.24–0.44]	0.001	0.145	[0.085, 0.247]	0.001
Waqooyi Galbeed	0.68	[0.55–0.84]	0.001	0.276	[0.195, 0.391]	0.001
Middle Shabelle	0.77	[0.60–0.98]	0.032	0.066	[0.023, 0.188]	0.001
Mudug	0.56	[0.43–0.73]	0.001	0.258	[0.152, 0.437]	0.001
Nugaal	0.65	[0.51–0.83]	0.001	0.382	[0.246, 0.593]	0.001
Sanaag	0.95	[0.76–1.20]	0.676	0.330	[0.218, 0.499]	0.001
Sool	0.87	[0.69–1.10]	0.259	0.368	[0.250, 0.543]	0.001
Togdheer	0.90	[0.72–1.12]	0.350	0.301	[0.195, 0.464]	0.001
